# Sex-dependent variation in cartilage adaptation: from degeneration to regeneration

**DOI:** 10.1186/s13293-023-00500-3

**Published:** 2023-04-05

**Authors:** Jhanvee Patel, Song Chen, Torey Katzmeyer, Yixuan Amy Pei, Ming Pei

**Affiliations:** 1 Stem Cell and Tissue Engineering Laboratory, Department of Orthopaedics, West Virginia University, 64 Medical Center Drive, PO Box 9196, Morgantown, WV 26506-9196 USA; 2 Department of Orthopaedics, The General Hospital of Western Theater Command, Chengdu, 610083 Sichuan China; 3 Perelman School of Medicine, University of Pennsylvania, Philadelphia, PA 19104 USA; 4 WVU Cancer Institute, Robert C. Byrd Health Sciences Center, West Virginia University, Morgantown, WV 26506 USA

**Keywords:** Sex, Cartilage, Degeneration, Regeneration, Stem cell therapy, Hormone

## Abstract

Despite acknowledgement in the scientific community of sex-based differences in cartilage biology, the implications for study design remain unclear, with many studies continuing to arbitrarily assign demographics. Clinically, it has been well-established that males and females differ in cartilage degeneration, and accumulating evidence points to the importance of sex differences in the field of cartilage repair. However, a comprehensive review of the mechanisms behind this trend and the influence of sex on cartilage regeneration has not yet been presented. This paper aims to summarize current findings regarding sex-dependent variation in knee anatomy, sex hormones’ effect on cartilage, and cartilaginous degeneration and regeneration, with a focus on stem cell therapies. Findings suggest that the stem cells themselves, as well as their surrounding microenvironment, contribute to sex-based differences. Accordingly, this paper underscores the contribution of both stem cell donor and recipient sex to sex-related differences in treatment efficacy. Cartilage regeneration is a field that needs more research to optimize strategies for better clinical results; taking sex into account could be a big factor in developing more effective and personalized treatments. The compilation of this information emphasizes the importance of investing further research in sex differences in cartilage biology.

## Highlights

- Evidence indicates males and females respond differently to cartilage degeneration and regeneration.
- Few review articles available provide comprehensive information referencing clinical and laboratory research.
- This review offers an update on sex-dependent variation in knee anatomy and sex hormones’ effect on cartilage degeneration and regeneration as well as potential application in stem cell therapy.
- Understanding sex differences in cartilage biology contributes to personalized treatment in cartilage diseases.

## Introduction

Adult cartilage defects present a challenge in orthopaedic medicine, as cartilage possesses limited intrinsic healing capacity. Onset of cartilage degeneration increases with age, leading to prevalent diseases such as osteoarthritis (OA) in the elderly. Cartilage degeneration is accompanied by pain and discomfort, hindering activities of daily living. These difficulties have prompted a mass accumulation of research in cartilage repair and regeneration. Stem cell-based therapy becomes a promising approach to healing cartilage in which patient-derived stem cells are rejuvenated and grown in vitro to be injected or implanted into cartilage defects. This therapy has the potential to reduce morbidity, mortality, and economic costs from complications of traditional surgical techniques [1].

Increasing evidence indicates that males and females differ in cartilage characteristics and risk of degeneration through sex-dependent gene expression [2], which necessitates consideration of sex differences when designing preclinical studies and clinical trials for cartilage repair [3]. For example, males have thicker articular cartilage and greater knee cartilage volume than females [4, 5]. Accordingly, females have a higher chance of developing OA than males, specifically in the knee after menopause [6]. Females also tend to experience more severe cases of knee arthritis and are more than three times more likely to be candidates for total knee or hip replacement surgery than males [5]. Despite increased scientific awareness of sex differences in cartilage development, degeneration, and repair, many studies continue to assign sex arbitrarily, as a comprehensive review of the differences in male versus female cartilage adaptation has not been presented. This review focuses on sex-dependent variation in cartilage degeneration and regeneration with an emphasis on stem cell-based therapies.

## Chondrocyte biology overview

This section provides a brief introduction of articular cartilage development and maintenance as well as associated signaling pathways.

### Development of articular cartilage

During fetal development and early neonatal life, articular cartilage does not have the same properties as adult cartilage and must progress through additional developmental stages. Early articular cartilage consists of matrix-poor tissue with an irregular and uneven distribution of cells; however, by 6 weeks of age, it increases in thickness through both chondrocyte hypertrophy and increases in matrix secretion [7]. As articular cartilage develops, it also establishes zonal organization consisting of the surface zone, medial zone, and deep zone. The surface zone consists of flat articular chondrocytes that produce and secrete lubricating proteoglycans encoded by the proteoglycan 4 (PRG4) gene for joint protection during movement. Both the medial and deep zones contain large, round, vertically oriented chondrocytes that excrete extracellular matrices. There is some uncertainty regarding the exact mechanisms underlying chondrocyte expansion in the neonate, but some studies suggest that PRG4-positive cells in the surface zone are responsible for cartilage growth and transformation [7]. Growth and differentiation factor 5 (GDF5), Wingless/integrated gene 9a (WNT9A), Doublecortin (DCX), SRY Box transcription factor 9 (SOX9), and ETS-related gene (ERG) are all genetic markers of cells in the interzone, which is the primitive joint during fetal development. The interzone consists of flat mesenchymal stem cells (MSCs) required for joint formation. By regulating expression of the above genes and others, the fate of interzone cells is determined [7]. For example, SOX9 is expressed throughout the interzone cells during early development, but by day 14, expression is limited only to the outer regions of the interzone and the flanking outer chondrocytes; the intermediate zone, on the other hand, ceases expression of type II collagen (COL2A1), leading to its involvement in the formation of cruciate ligaments. Finally, transforming growth factor beta (TGFβ) receptor 2 (TGFBR2)-expressing interzone cells are found only in the dorsal and ventral regions of joints in mature cartilage, indicating that expression is either induced in these regions or deleted in other regions to form adult articular cartilage [8]. GDF5 is expressed in all of these cells and, therefore, is a broad marker associated with multiple cell types within the interzone that give rise to not only articular cartilage, but also synovial tissue and intra-joint ligaments [7]. However, interzone cells alone are not sufficient for normal articular cartilage development. Interzone cells were found to be mitotically quiescent, while flanking cells of non-interzone origin were mitotically active, indicating that the underlying proliferative cells that get recruited by interzone cells to the flank area play a major role in the thickening of developing articular cartilage [7].

### Maintenance and repair of articular cartilage

Articular cartilage in the adult is considered a permanent tissue, meaning it has little-to-no turnover throughout adult life. This property differs from cartilage in other locations, such as growth plate cartilage. ERG expression persists only in the superficial cells of the articular cartilage after 6 months of age and is responsible for the permanent quality of articular cartilage. In the case of *ERG* deficiency, Friend leukemia integration 1 (*FLI1*), another transcription factor, provides redundancy to maintain the permanent nature of articular cartilage [7]. Although articular cartilage exhibits limited capacity for cell turnover and regeneration, there are progenitor cells that exist within fully developed articular cartilage; however, under normal conditions, these cells do not proliferate in the case of injury or degeneration, such as in OA [7]. These progenitor cells have been targeted as a mechanism of OA treatment through the surgical movement of these cells to the cartilage surface and induction of their proliferation. Several proteins and hormones have been indicated in the maintenance and protection of articular cartilage, including parathyroid hormone (PTH), PTH related protein, and PRG4; in addition, the Hedgehog signaling pathway as well as A disintegrin and metalloproteinase with thrombospondin motifs 5 (ADAMTS5) and metalloproteinase have been indicated in cartilage pathogenesis, as ablation and elimination have shown increased resistance to OA development in mice [7].

Signaling pathways involved in articular cartilage anabolism and/or anti-catabolism include TGFβ1, insulin-like growth factor I (IGFI), hypoxia-inducible factor (HIF) 1 alpha (HIF1α), and bone morphogenetic protein 7 (BMP7) [9]. Catabolic signals involve interleukin 1 (IL1), IL6, HIF2α, and fibronectin fragments. Conflicting information exists regarding the role of fibroblast growth factor 2 (FGF2) in articular cartilage maintenance. These mechanisms are highlighted throughout the discussions in the “Inflammatory biomarkers” and “Molecular mechanisms” sections [9].

## Sex differences in knee anatomy and cartilage degeneration

There are observed anatomical differences in cartilage growth and knee structure between sexes, which could contribute to the sex-related differences in cartilage degeneration [10]. The rate of cartilaginous degeneration in an individual is multifactorial, and several mechanisms influencing risk of OA and cartilage injury are sex-dependent in nature [10].

### Knee anatomy and articular cartilage thickness and degeneration

In the distal femur and proximal tibia, the mean aspect ratio (mediolateral distance versus anteroposterior distance) of the male femora is larger than the female femora [11]; the plateau aspect ratio (tibial medial anteroposterior dimension versus tibial mediolateral dimension) of the male is also larger than the female [12]. With a high degree of variation between individuals, sex-specific designs of total/unicompartmental knee arthroplasty have been developed to accommodate anatomical differences between sexes [11, 13].

In healthy children, cartilage thickness in the knee differs significantly between sexes, with girls having thinner cartilage than boys [14, 15]. Weight, height, and body mass index (BMI) contribute to cartilage thickness, but age is the leading contributor among school age children [14]. Physical activity benefits tibial cartilage volume [16]. In adults, males have substantially higher cartilage thickness and volume than females [17]. Both sexes exhibit a clear decrease in cartilage with increasing age, but the decrease is more drastic for females [15, 18]. Females have a higher incidence rate of obesity in most countries, which has been linked to increased risk of OA through increased weight on joints [19]. A high BMI was closely related to both knee and hand OA rather than hip OA [20]. Weight reduction becomes an important part of OA treatment [21]. Similar to obesity, ethnicity is also a co-variable that influences OA incidence and severity. Elderly Chinese women in Beijing have a higher knee OA incidence than women in Framingham, Massachusetts; however, the incidence in men was comparable [22]. African–Americans have a higher prevalence of knee OA than Caucasians in the U.S., but this rate may vary according to sex [23]. Despite the progress in some racial differences in OA incidence and severity, more attention should be put on under-studied racial and ethnic groups and joint groups (e.g., foot, spine), emphasizing potential analytical factors, including but not limited to genetic, anatomical, environmental, and biomechanical features [23].

### Inflammatory biomarkers

In both healthy individuals and OA patients, females have higher levels of inflammatory biomarkers [24]. For example, healthy women tend to have higher serum levels of leptin, an adipokine with pro-inflammatory properties [25]. As an essential hormone for bone development, high leptin levels in older adults are associated with lower cartilage volumes, and therefore, this hormone is thought to contribute to the increased OA susceptibility in women [25, 26]. Extreme obesity caused by impaired leptin signaling leads to changes in subchondral bone morphology, but does not increase the incidence of knee OA, suggesting that obesity due to leptin-impaired signaling is insufficient to induce systemic inflammation and knee OA in female C57BL/6J mice [27]. As a link between obesity and OA [28], the use of leptin might be a potential approach for therapy in bone and joint diseases [29], especially for obese patients. In OA patients, pro-inflammatory chemokine CC chemokine ligand 3-like-1 (CCL3L1) is also found in higher levels in females than males, along with several inflammatory cytokines [30]. Interestingly, females with OA also tend to have higher serum levels of anti-inflammatory adipokines, such as apelin and adiponectin [31], suggesting that sex differences in cartilage degeneration are not perfectly aligned with the level of inflammation in the joint.

When comparing the synovial fluid composition in male and female OA patients, women had less 25-hydroxyvitamin D3 [25(OH)D3], a metabolite of Vitamin D that protects against cartilage degeneration [32], which may explain why men with OA typically have lower inflammatory markers. Dehydroepiandrosterone (DHEA), the precursor for both estrogen and testosterone, is one agent with promising anti-inflammatory effects for both sexes. DHEA injection into arthritic joints of male and female rabbits resulted in decreased expression of inflammatory response elements, such as matrix metalloproteinase 3 (MMP3) and increased tissue inhibitor of matrix metalloproteinase 1 (TIMP1), indicating that DHEA treatment may protect against further cartilage degradation in both men and women by reducing inflammation [33]. Interestingly, male rats have higher sensitivity to anti-inflammatory agents, such as parecoxib and dexamethasone, while female rats exhibited higher levels of IL6 after dexamethasone treatment, which likely explains the limited response of females to anti-inflammatory treatment [34]. IL1, another catabolic pro-inflammatory cytokine, may be upregulated by FGF2, which is correlated with expression of estrogen receptors (ERs) [35, 36]. According to a study published in Clinical and Experimental Rheumatology, the articular cartilage of female rats was found to be more sensitive to IL1-mediated inhibition of proteoglycan synthesis, increasing risk of cartilage damage [35].

### Extracellular matrix biomarkers

It has been observed that there are differences between sexes on extracellular matrix (ECM) biomarker expression following the onset of cartilage degeneration. Variations in ECM composition can significantly influence cell behavior. In healthy females, ECM of articular cartilage is likely to have higher spontaneous loss of glycosaminoglycans (GAGs) along with reduced levels of proteoglycan and collagen when compared to males [35]. Structural change to ECM proteins is a classic indicator of early OA, supporting the clinical findings of female predisposition to OA development [37]. Another sex disparity in OA is the rate of collagen turnover. Female OA patients tend to have genetic upregulation of type I collagen, indicating that OA females lose collagen at a faster rate than OA males and, therefore, require more collagen transcription to compensate [38]. Female chondrocytes are also more susceptible to the inhibition of proteoglycan production by IL1, indicating that females are less likely to synthesize new proteoglycan than males [35].

Another OA biomarker found in synovial fluid, cartilage acidic protein 1 (CRTAC1), is induced by inflammatory cytokines, such as IL1 [39]. As female chondrocytes are more sensitive to IL1, CRTAC1 tends to exist at higher levels in the female population than in males [39]. Therapeutic research using murine models is beginning to look into the feasibility of *CRTAC1* gene knockout in the prevention of female OA development [39].

When comparing men and women with clinical OA, C-terminal telopeptide of type I collagen (CTX-I) and, to a lesser degree, CTX-II, are both found in higher serum concentrations in females [40]. CTX is a product of cartilage degradation, so higher serum CTX levels indicate increased damage to articular cartilage; in addition, levels of CTX-II are associated with pain intensity, suggesting that OA females likely perceive more pain related to their cartilage loss than males with the same condition [41]. Increased serum CTX also illustrates the predisposition to OA susceptibility in females found in clinical studies.

Serum values of cartilage oligomeric matrix protein (COMP) also vary based on sex, with healthy Caucasian males having higher serum COMP on average compared to healthy Caucasian females [42]. COMP expression also varies by ethnicity, as COMP levels were found to be higher in African–American women than Caucasian women with corresponding ages; no significant COMP differences were found between African–American men and women or between Caucasian and African–American men [42]. In all groups, increasing age and BMI were directly associated with elevated serum COMP [42]. Increased COMP levels predict subsequent cartilage loss, but the degree of association is only around 60 percent; nevertheless, elevated serum COMP is a mild predictor of cartilage degradation, so this finding contrasts from the majority of other degenerative biomarkers in which women typically present with higher levels [43]. There is some contradictory evidence, however, regarding the sex differences of COMP levels. COMP levels increase acutely with physical activity, and women with thinner anterior femoral cartilage have greater resting COMP levels [44].

## Protective effect of sex hormones on cartilage degradation

Cartilage growth regulation is complex. Several factors act on chondrocytes during their proliferation and differentiation, such as IGF-I and -II, FGF2, TGFβ, epidermal growth factor (EGF), platelet-derived growth factor (PDGF), and sex hormones (estrogens and androgens) [45]. Estrogens promote chondrocyte proliferation, and androgens affect chondrocyte proliferation via conversion to estrogen by aromatase [45]. Male and female differences in these factors, especially sex hormones, could be crucial for developing specific treatments targeting cartilage degradation (Table 1).

**Table 1:** Impact of sex hormones on cartilage degeneration

Hormones	Study design	Treatment	Analysis	Results	Implications	References
T/DHT	In vivo; male murine model	ORX versus non-ORX	Histologic analysis of knee cartilage explants	ORX males had lower OA scores and significantly reduced OA severity	Male hormones may accentuate OA severity in this model	[111]
	In vitro; murine chondrocytes from costochondral cartilage	Cultured with media containing 10^–11^ M T or DHT	ALP analysis for differentiation and LSS analysis for proliferation	Male growth zone chondrocytes showed dose-dependent increases in thymidine incorporation and increased ALP activity; male or female resting zone chondrocytes showed no changes in thymidine incorporation; male resting zone chondrocytes or female chondrocytes from any regions showed no changes in ALP activity	Only male cells have a physiologic response to T and DHT treatment	[230]
T	In vivo; arthritic rat model	ORX followed by DHT treatment	Clinical OA evaluation, lysosomal activity, TBARS level (indicative of membrane damage)	Castrated rats exhibited elevated lysosomal activity and higher TBARS; DHT treatment after castration lowered TBARS level; castrated rats mounted a severe immune response that was reduced upon DHT application	T and DHT exhibit anti-inflammatory effects at the joint in both males and females	[94]
	In vivo; human patients with severe knee OA	Unilateral TKR	Serum T levels, knee radiograph, and WOMAC pain/function analysis 6–8 weeks after surgery	On the operative knee, higher T levels were associated with less pain in both sexes; in the non-operative knee, higher T was associated with less disability in women	T is negatively correlated with joint disability in women	[196]
E2	In vivo; female murine model	OVX versus non-OVX	Histologic analysis of knee cartilage explants	Lesions seen in OVX mice were significantly more severe than those seen in control females, but less severe than those seen in control male mice	E2 loss is associated with cartilage loss/OA; suggests E2 may be chondroprotective	[111]
	In vitro; human female articular chondrocytes from OA patients	E2 exposure during proliferation-stock solutions of 10^–1^ M E2 in absolute ethanol diluted stepwise. Controls in 0.1% ethanol	qPCR, Western blotting, immunofluorescence	E2 suppressed MMP13 expression in female human articular chondrocytes	E2 may be chondroprotective	[76]
	In vitro; bovine model articular chondrocytes of unspecified sex	Grown in media dosed with E2 (concentrations of 0 M, 10^–11^ M, 10^–10^ M, 10^–9^ M, 10^–8^ M, 10^–7^ M, 10^–6^ M, 10^–5^ M, and 10^–4^ M), then treated with H_2_O_2_ on day 7 for radical generation	Toxicity assay, morphology, and DNA quantification via fluorometer	Chondrocytes incubated with E2 showed better morphology than control after radical treatment; E2 chondrocytes released LDH at 7% while control released it at 61%	E2 is protective against free radical damage in chondrocytes	[231]
	In vivo; female rabbit knee articular cartilage	Total RNA extraction of pregnant versus control rabbits	RT-PCR	Significant decrease seen in mRNA levels for type II collagen, biglycan, collagenase, TIMP1, TNFα, iNOS in pregnant rabbits	Pregnancy, which is associated with steady rises in estrogen, is associated with depression of mRNA expression in cartilage	[232]
	In vivo; human female articular cartilage observational study	ERT current users, former users, and non-users	Examinations, anteroposterior weight-bearing radiographs	Females who never used ERT had worse OA than females who used ERT	ERT could have a protective effect on cartilage	[194]
	In vivo; human female articular cartilage	ERT greater than or equal to 5 years	T1 weighted fat suppressed MRI of knees	Higher tibial cartilage volume found in ERT users than non-users	ERT may prevent loss of knee articular cartilage	[193]
	In vivo; murine model	Mice with ERα inactivation versus wild-type; OVX followed by treatment with E2 or placebo, then induced with antigen-induced arthritis	Histology, flow cytometry, T-cell proliferation assay	In wild-type mice (that have estrogen receptors), E2 treatment decreased synovitis and joint destruction; E2 did not affect ERα knockout mice	E2 works through ERα to protect against cartilage damage	[188]
PG	In vivo; murine knee chondrocytes	TRPV knockout mice versus wild-type controls	Quantification of chondrocytic calcium signaling with fluorescence imaging	TRPV knockout males demonstrated the most severe cartilage erosion; no significant difference found between TRPV knockout females and wild-type females at any timepoint	PG exposure may decrease TRPV4 expression	[233]
	In vitro; human tracheal epithelial cells	Media supplemented with PG	RT-PCR, Western blot, luciferase assay, calcium measurements via fluorescence imaging	PG-treated cells exhibited downregulation of TRPV4 channels	PG mediates the downregulation of TRPV4	[178]
DHEA	In vivo; rabbit model with unilateral ACL transection	DHEA injection into knee joint at dose of 100 µM DHEA dissolved in DMSO	Histological evaluation, RT-PCR	Significant decrease in severity of lesions was seen after DHEA injection and MMP3 expression was downregulated in DHEA group	DHEA may be protective against the development of OA in both sexes	[33]
T, E2, PG	In vivo; human knee cartilage	Vitamin D treatment	Sex hormone assay	High T levels correlated with lower pain levels in females, high E2 levels correlated with lower grade bone marrow lesions in females, high E and progesterone levels correlated with lower effusion–synovitis volume, high progesterone correlated with higher cartilage volume	Low levels of T, E2, and progesterone are associated with worsening OA in females, but not males	[234]

*ACL* anterior cruciate ligament, *ALP* alkaline phosphatase, *DHEA* dehydroepiandrosterone, *DHT* dihydrotestosterone, *DMSO* dimethylsulfoxide, *E2* 17-ß estradiol, *ER* estrogen receptor, *ERT* estrogen replacement therapy, *H*_*2*_*O*_*2*_ hydrogen peroxide, *iNOS* inducible nitric oxide synthase, *LDH* lactate dehydrogenase, *LSS* liquid scintillation spectroscopy, *MMP13* matrix metalloproteinase 13, *MRI* magnetic resonance imaging, *OA* osteoarthritis, *ORX* orchiectomy, *OVX* ovariectomy, *PG* progesterone, *qPCR* quantitative real-time polymerase chain reaction, *RT-PCR* reverse transcription polymerase chain reaction; *TBARS* thiobarbituric acid reactive substances, *TIMP1* tissue inhibitor of matrix metalloproteinase 1, *TRPV* transient receptor potential vanilloid, *T* testosterone, *TKR* total knee replacement, *TNFα* tumor necrosis factor α, *WOMAC* Western Ontario and McMaster Universities Osteoarthritis Index

### Effect of estrogen on cartilage

Estrogen is a steroid hormone existing in the body in three different subtypes: estrone (E1), estradiol (E2), and estriol (E3) [46]. E2 is the most prevalent and has the highest bioactivity. These estrogen subtypes signal through three main ERs: nuclear ERα and ERβ, and membrane G-protein coupled ER (GPER/GPR30) [46]. Availability of aromatase, the enzyme responsible for conversion of testosterone to estrogen, directly corresponds to estrogen levels in both males and females. Granulosa cells in the female ovary contain aromatase, as well as bone, breast, brain, and adipose tissue in both sexes. Therefore, obesity increases the availability of estrogen in both sexes. Consideration of aromatase availability must be taken into consideration while evaluating the impact of estrogen on cartilage [47].

#### Estrogen receptors and cartilage

In 1999, Ushiyama et al. identified the gene expression of both ERα and ERβ in human articular chondrocytes [48]. They found that, despite the fact that women have higher estrogen levels, men show a significantly higher level of gene expression for both ER paralogs than women. All women in the study were postmenopausal and had never had estrogen replacement therapy (ERT), which is linked to decreased estrogen levels. Expression of ERα in articular cartilage decreases with age, which can be linked to cartilage degeneration and increased OA severity [49]. Selective ER modulators have clinical utility in the context of osteoporosis, ER^+^ breast cancer, and other estrogen-related pathologies to mediate stimulation and/or antagonism of site-specific ERs in the body. Raloxifene, often prescribed for osteoporosis, has been observed to activate ERs and the extracellular signal-regulated kinase 1/2 (ERK1/2) signaling pathway in human chondrocytes, preventing tumor necrosis factor alpha (TNFα)-induced caspase-3-dependent apoptosis [50]. In addition, estrogen via an ERβ-dependent mechanism inhibits cell proliferation and ERα expression, while estrogen via an ERβ-independent mechanism regulates chondrogenesis [51]. ERβ deficiency has been documented to increase condylar growth in female mice by inhibiting the turnover of fibrocartilage [52]. Overall, studies point to increased ERα expression having both a chondroprotective effect and an inhibitory role in ERβ expression during chondrogenesis.

The estrogen‑related receptor (ERR) family of orphan nuclear receptors related to ERα, composed of ERRα, ERRβ, and ERRγ, has been shown to potentially play a significant role in OA pathogenesis [53, 54]. Despite sharing sequence homology to the ERα and ERβ, ERRα is unable to bind estrogen [53]. ERRα could have dual contrasting roles in the induction and progression of OA. ERRα is essential for cartilage formation by regulating its target gene *SOX9* expression [54]. One study has shown, similar to its effect on ERα, 17β-E2 increased mRNA and subsequent protein expression of ERRα, which in turn led to an increase in *SOX9*, *GDF5*, and *CYP19A1* (aromatase) during in vitro mandibular condylar chondrocyte cultivation [55]. SOX9 and GDF5 contribute to chondrocyte proliferation, differentiation, and maturation. When XCT790, a synthetic inverse agonist of ERRα, was used to inhibit ERRα expression, the proliferative capacity of the mandibular condylar chondrocytes was reduced [55]. Furthermore, knockdown of ERRα has resulted in impaired expression of genes including *SOX5*/*SOX6*/*SOX9*, *COL2A1*/*COL10A1*, and *RUNX2* (runt-related transcription factor 2) and ectopic expression of SOX9 rescued defective formation of cartilage, indicating that ERRα is involved in chondrocyte growth by regulating SOX9 expression [56]. However, ERRα-mediated degradation of cartilage has also been observed. Increased expression of ERRα has been linked with IL1β treatment in human OA chondrocytes via the PGE2 (prostaglandin E2)/cAMP (cyclic Adenosine 3′,5'-monophosphate)/PKA (protein kinase A) signaling pathway [57]. ERRα could upregulate IL1-induced *MMP13* expression in OA chondrocytes. In addition, XCT790 decreased *MMP13* gene level [57]. These results point to ERRα involvement in IL1β-mediated OA cartilage degradation and loss.

Of the ERR subtypes, ERRγ has significantly increased expression in humans and various models of mouse OA cartilage. Overexpression of ERRγ in cartilage is connected to chondrodysplasia and reduced chondrocyte proliferation [57]. ERRγ contributes to cartilage destruction through the IL6-mediated mitogen-activated protein kinases (MAPK), such as the ERK1/2 pathway [58]. As a downstream transcription factor of ERK1/2, upregulation of ERRγ leads to ECM degradation and angiogenesis in osteoarthritic temporomandibular joints (TMJ) [59]. Overexpression of ERRγ in chondrocytes directly upregulates MMP3 and MMP13 expression. Moreover, GSK5182, a small-molecule ERRγ inverse agonist, has promising therapeutic potential by inhibiting pro-inflammatory cytokine-induced catabolic factors [60]. As ERRγ is involved in the catabolic modulation of OA pathogenesis, it has strong potential to be a therapeutic target for OA [61].

During early puberty, GPER1/GPR30 positively regulates chondrocyte proliferation at the growth plate, contributing to the longitudinal growth of long bones [62]. GPER1/GPR30 has been observed to alleviate mechanical stress-mediated apoptosis of chondrocytes in OA through suppression of Piezo1, a mechanosensitive ion channel ubiquitously expressed in adipose tissue that, when upregulated, has been linked to chondrocyte apoptosis [63]. E2 works with GPER1/GPR30 to suppress acid-sensing ion channel 1a (ASIC1a) and ASIC1a can overregulate intracellular calcium levels, resulting in articular chondrocyte damage [64]. E2’s suppression of the channel protects rat cartilage with adjuvant arthritis from acidosis-mediated injury and autophagy [64].

#### Estrogen and cartilage

Estrogen is an attractive candidate for cartilage engineering due to evidence that local estrogen production is crucial for chondrocyte proliferation and protection from spontaneous cell death [65]. Chondrocytes have been observed to be capable of both in vivo and in vitro estrogen synthesis [66], which appears to increase *COL2A1* gene expression [67].

Growth factors, such as TGFβ, BMP, FGF, PDGF, growth hormone (GH), and IGF-I, are integral for cartilage development and healing. Observations suggest that E2 may interact with the synthesis and secretion of these growth factors, specifically TGFβ and IGF-I. TGFβ1 has been observed to be a modulator in 17β-E2 activity on costochondral chondrocytes from female rats in a sex-specific manner [68]. IGF promotes the production of matrix as well as the proliferation and inhibition of apoptosis in chondrocytes [69]. IGF regulation could be influenced by estrogen action [70]. Research demonstrates that E2 has an indirect effect of priming IGF-I activity in cartilage metabolism [71]. The interaction between the IGF-I receptor and ERα has been observed to promote proliferation and suppress inflammation in nucleus pulposus cells [72].

Catabolic cytokines such as IL1 have detrimental effects on the composition and mechanical properties of articular cartilage. Research suggests estrogen deficiency can increase cytokine receptor numbers and cofactors of cytokine action, which enhances cell response to cytokines [73]. β-Ecdysterone, an estrogen analog, has demonstrated anti-apoptosis and anti-inflammation ability in IL1β-induced rat chondrocytes [74].

Estrogen has been documented to suppress MMPs. MMP1, MMP3, and MMP13 are intimately involved in the process of articular cartilage degeneration [75]. One study observed 17β-E2 suppressed MMP13 expression in human articular chondrocytes [76]. The use of 17β-E2 in physiological doses can improve the MMP and TIMP imbalance in articular chondrocytes, suggesting a potential chondroprotective effect of hormone replacement therapy [77, 78]. Current findings demonstrate that estrogen can be modulated to reduce the effect of reactive oxygen species (ROS). In rat nucleus pulposus cells, the interaction between E2 and ER has interfered with the ROS/nuclear factor kappa-B (NF-κB) pathway, reducing TNFα-induced premature senescence [79]. Pretreatment with 17β-E2 not only decreased acid-induced damage, it also inhibited apoptosis and helped to restore mitochondrial function. Specifically, studies have shown that 17β-E2 is capable of decreasing levels of ASIC1a through the ERα and the autophagy–lysosomal pathway [80].

While estrogen can play a protective role in cartilage through the many factors discussed above, it has been documented to have detrimental effects as well. 17β-E2 stimulation has also resulted in the loss of ECM and increased expression of TNFα, IL1, HIF2α and its downstream OA-related cytokines [MMP13, vascular endothelial growth factor (VEGF), and type X collagen] in primary condylar chondrocytes via ERβ [81]. Estrogen has been reported to be chondrodestructive in animal models; specifically, increased activity of ERs has been suggested as a factor in initiating osteoarthritic changes in a rabbit model [82]. High E2 concentration has been linked to increased IL1β stimulated proteoglycan degradation and MMP production in chondrocytes [83]. Interestingly, E2 has been observed to reduce nerve growth factor (NGF) expression in chondrocytes significantly, even after stimulation by TGFβ1 or IL1β, indicating estrogen can play a role in regulating NGF, which is integral to the development of OA pain, suggesting that E2 is associated with decreased OA pain [84]. The contradictory results make it difficult to understand the role estrogen plays in cartilage degradation and further demonstrates more research is needed.

### Protective effect of androgens on cartilage

Androgens are steroid hormones with the most prevalent being testosterone, dihydrotestosterone (DHT), and androstenedione. In addition, androgens are the precursors for estrogen. Like estrogen, research suggests that androgens play a role in cartilage protection [85]. Androgen levels are high in males and low in females, which could be a reason why males have less risk of OA. However, 17β-E2 has been observed to have a greater impact in chondrocyte functionality and gene expression profiles, which is particularly apparent in chondrocytes from females [86]. Regardless, understanding the relationship between chondrocytes and androgens is a crucial step in determining OA risk differences between males and females.

#### Androgen receptors and cartilage

Androgen receptor (AR) is a steroid hormone receptor that influences the transcription of androgen-responsive genes by binding their respective DNA sequences [87]. Moreover, AR can affect cell physiological activities, such as proliferation, apoptosis, and migration [88]. AR is expressed in human primary articular chondrocytes [89]. Testosterone receptors have been discovered in rat chondrocytes from growth zone and resting zone cartilage in both sexes [90]. Additionally, in rabbits, AR overexpression has resulted in a reduced apoptosis rate and has maintained the phenotype of chondrocytes through inhibition of the mammalian target of rapamycin (mTOR) pathway to improve autophagy [88].

#### Androgen and cartilage

Androgens are related to cartilage tissue maintenance. Testosterone at physiological concentrations increases chondrogenic potential of chondrogenic progenitor cells in male arthritic tissue in vitro [91]. In male intervertebral disc (IVD) cells, testosterone has effectively enhanced chondrogenesis in vitro but does not affect female IVD cells or mesenchymal stem cells (MSCs) from either sex similarly [92]. In chondrocytes of mice and rabbits, testosterone stimulates growth and local production of IGF-I [71, 93], suggesting that testosterone has an indirect priming effect on the response of chondrocytes to IGF-I.

Studies have also shown DHEA has a protective role against OA, specifically with inflammation [33, 94, 95]. A rabbit study concluded that DHEA has a cartilage-protecting effect during OA development following bilateral anterior cruciate ligament transection [96]. In male and female mice, DHEA treatment has demonstrated the ability to delay onset and decrease the severity of collagen-induced arthritis [97]. In human osteoarthritic knee chondrocytes, DHEA treatment has been shown to significantly reduce *MMP1* but increase *TIMP1* gene expression and protein levels [98]. In rats with synovial arthritis, DHT treatment has reduced TNFα and MMP2 levels [99]. These data indicate that DHEA is associated with reduced inflammation and modulation of collagen breakdown in both rats and humans.

Androgens could play a role in cartilage inflammation through cytokines. Androgens stimulate articular cartilage integration. Low concentrations of IL1β could influence this effect favorably [100]. In female animals, testosterone has been shown to have a protective influence on IL1-induced cartilage breakdown [101]. Moreover, testosterone has decreased the effect of IL1 on both proteoglycan loss and synthesis, which are crucial parts of cartilage ECM. Androgens also have demonstrated a protective role in the development of adolescent idiopathic scoliosis, potentially by inhibiting IL6-induced abnormal chondrocyte development [102]. The mechanisms behind the association between androgens and cytokines could be numerous, involving direct immunomodulatory effects and interaction of glucocorticoid response to inflammation [100].

## Effect of sex hormone on subchondral bone and adjacent synovium

Strong evidence supports a connection between subchondral bone changes and cartilage damage and loss [103]. Estrogen has been investigated as an influence in this relationship, because it has been documented to increase cartilage alteration [104]. In OA, increased production of synovial fluid results in swelling of the synovium [105]. Understanding the influence of sex hormones on these structures is crucial to understanding the differences in male and female OA prevalence.

### Protective effect of sex hormones on subchondral bone

Multiple studies point to estrogen modulating OA by increasing subchondral bone structure. Bone remodeling has been connected to estrogen depletion by ovariectomy, affecting the subchondral trabecular bone of joints [106, 107]. Estrogen deficiency led to subchondral bone resorption and articular cartilage degeneration in an ovariectomized (OVX) rat model of postmenopausal OA [108]. Estrogen replacement treatment in a cynomolgus macaque model protects subchondral bone mass from remodeling [109]. Estrogen treatment in elderly women has been observed to lessen subchondral bone weakening in the OA knee [110]. ERs are also commonplace in bone tissue and help to regulate bone turnover, which is relevant to OA pathophysiology. In murine models, ERα knockout has led to development of larger osteophytes and a thinner lateral subchondral plate [111, 112]. No studies currently exist examining the influence of androgen on subchondral bone.

### Effect of sex hormones on adjacent synovium/synovial fluid

Synovial fluid is made up of a myriad of cellular metabolites, one of which are extracellular vesicles (EVs). Exosomes are 40–100 nm diameter packaged vesicles comprised of lipid, protein, and small RNA [113]. Synovial fluid-derived EVs have demonstrated the ability to change microRNA (miRNA) cargo with sex-specific alterations. Within the synovial fluid of OA patients, exosomal miRNA content can be altered, and in females, there exist some estrogen responsive miRNAs that are capable of targeting toll-like receptor (TLR) pathways [114]. In female OA EVs, studies have observed increases in haptoglobin, orosomucoid, and ceruloplasmin levels and a decrease in apolipoprotein; in male OA EVs, β-2-glycoprotein and complement component 5 proteins have been observed to increase, and Spt-Ada-Gcn5 acetyltransferase (SAGA)-associated factor 29 has decreased [115]. The sex-specific alteration in synovial fluid EV protein content with OA patients could be a mechanism behind the high OA prevalence and severity in women.

In the TMJ, fibroblast-like type B synoviocytes could be affected by expression of ERα-immunoreaction, indicating that TMJ can be influenced by estrogen and resulting in higher prevalence of temporomandibular disorders in females than males [116]. Interestingly, compared to ERα, normal human synovia regularly expresses large amounts of ERβ [117]. Arthritic synovium also demonstrates expression of ERs, linking estrogen as a modulator in synovial inflammation [118]. Estrogens have been shown to aggravate TMJ inflammation and pain, potentially by amplifying the expression of cadherin-11 and release of pro-inflammatory cytokines in synoviocytes [119]. It has also been suggested that E2 aggravates TMJ inflammation by the NF-κB pathway, which similarly results in pro-inflammatory cytokine release [120]. In a rat study, male TMJ particularly was found to be an estrogen target especially for ERα [121]. Additionally, estrogen regulates the IGF system and cytokines, which act in the synovial fluid [70]. In addition to the prophylactic impact estrogen has through inhibiting the synovial inflammation and articular cartilage degeneration seen in OA, estrogens are suspected to also partially regulate sensory neuropeptide expression in the synovium of experimental OA models (anterior cruciate ligament transection rat models), for instance substance P and calcitonin gene-related peptide [122].

Chondrocytes from human OA synovium and intra-articular injection in rabbit OA knee joints have demonstrated suppressed *MMP3* expression and enhanced *TIMP1* level when treated with DHEA, indicating a protective role of androgens in cartilage degradation and synovial inflammation [33, 98]. DHEA has also been observed to have a protective effect in the synovial tissue of TMJ, potentially through increasing fibromodulin formation, which could prevent IL1β-induced inflammation and TGFβ1-induced hyperplasia of fibrous tissue [123].

## Molecular mechanisms

**Fig. 1:**
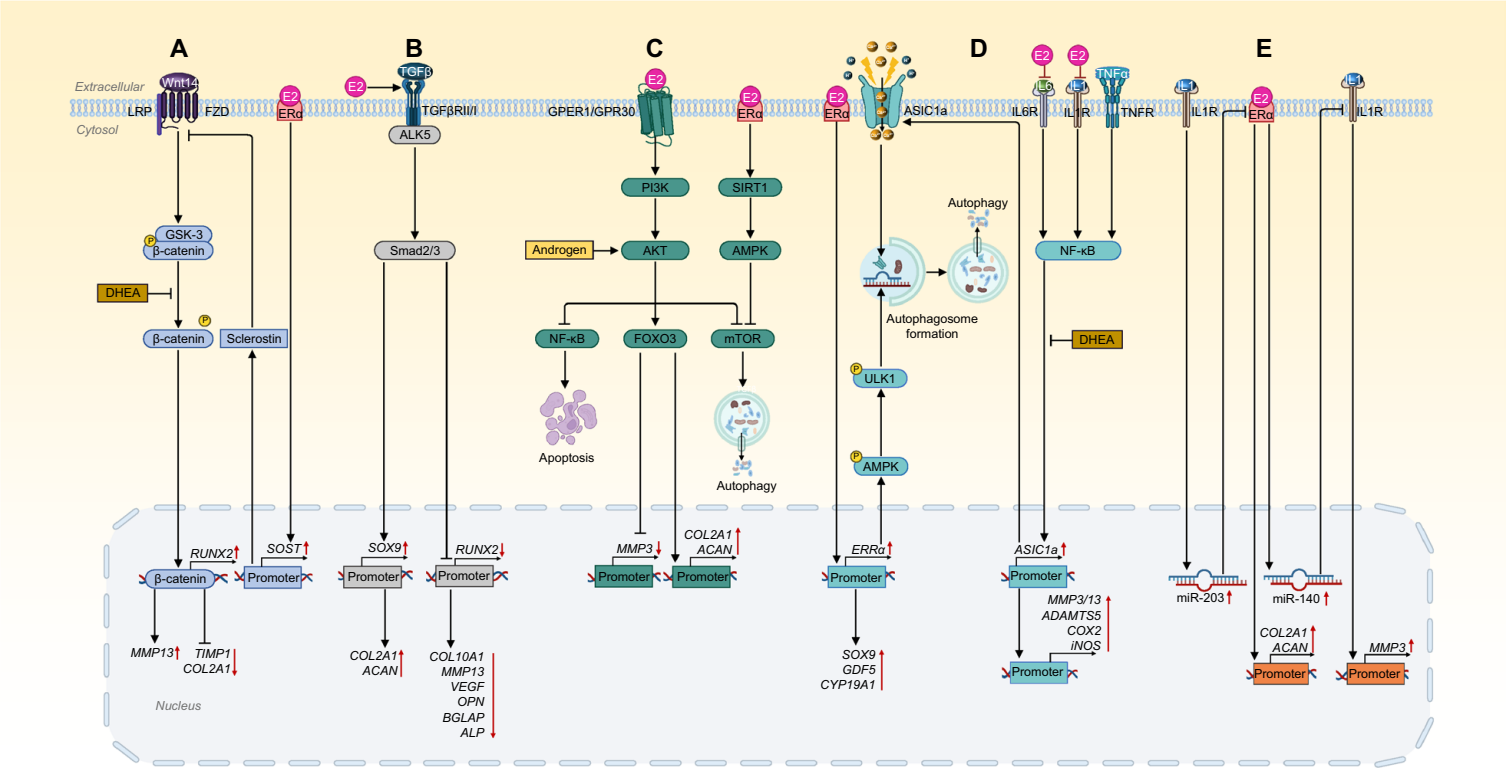
Main signaling pathways of sex hormones on chondrocytes. Wnt signaling pathway (**A**): Wnt14 binding inhibits the phosphorylation of β-catenin by GSK3, and the unphosphorylated β-catenin then travels into the chondrocyte nucleus to act as an OA phenotype transcription factor [9]. E2 upregulates the expression of *SOST*, which codes for an inhibitor of Wnt14 called sclerostin [130]. DHEA decreased the expression of β-catenin, resulting in upregulation of *MMP13* and downregulation of *TIMP1* and *COL2A1* [131]. TGF-β signaling pathway (**B**): E2 upregulates the expression of ALK5 receptors, promoting *ACAN* and *COL2A1* production and inhibiting *COL10A1*, *MMP13*, *VEGF*, *OPN*, *BGLAP*, and *ALP* expression [9, 135, 136]. Cellular energy and survival related pathways (**C**): PI3K/AKT signaling pathway is downregulated in human cartilage tissues with OA or in OA-like chondrocytes exposed to IL1, TNFα [153, 154]. E2 could function through the PI3K/AKT/NF-κB pathway by inhibiting chondrocyte apoptosis [229], through the PI3K/AKT/FOXO3 pathway by downregulating *MMP*3 expression and preventing ECM degradation [156]. Upregulated PI3K/AKT/mTOR in OA cartilage is linked to decreased expression of autophagy-related genes [158]. Overexpression of androgen has been shown to promote chondrogenesis and prevent degradation and apoptosis, potentially through mTOR-related signaling inhibition [88]. In addition, E2 inhibited autophagy upregulation to protect chondrocytes via the SIRT1-mediated AMPK/mTOR pathway [165]. Acid environment and cellular inflammation related pathways (**D**): E2 can increase the mRNA and protein expression levels of *ERRα*, which in turn led to an increase in *SOX9*, *GDF5*, and *CYP19A1.* Through the ERRα–AMPK–ULK1 signaling pathway, E2 could support autophagy–lysosome pathway-dependent ASIC1a protein degradation and defend against acidosis-induced cytotoxicity [168]. IL1/6 and TNFα activate NF-κB signaling pathways through receptor binding and ultimately help to upregulate expression of ASIC1a. Activation of ASIC1a could aggravate the effects of IL1/6 and TNFα on ECM metabolism by increasing *MMP3*/*13* and *ADAMTS5* mRNA expression in articular chondrocytes [166]. Extracellular acidification activates ASIC1a, which ultimately leads to the autophagy of articular chondrocytes [167]. Low levels of E2 have been observed to inhibit IL1-induced proteoglycan degradation, downregulating cartilage degeneration [83]. DHEA has been shown to play its protective role against cartilage degeneration through regulation of *MMP3*, *TIMP1*, *IL1, COX2*, and *iNOS* gene expression [33, 169]. MicroRNA related pathways (**E**): IL1 stimulation increased miR-203 expression [171]. MiR-203 directly targets ERα, followed by downregulation of *ACAN* and *COL2A1* [170]*.* The estrogen/ER/miR-140 pathway inhibited IL1-induced cartilage matrix degradation [76]. *LRP* low density lipoprotein receptor-related protein, *FZD* frizzled receptor, *GSK3* glycogen synthase kinase 3, *DHEA* dehydroepiandrosterone, *RUNX2* runt-related transcription factor 2, *MMP13* matrix metalloproteinase 13, *TIMP1* tissue inhibitor of matrix metalloproteinase 1, *COL2A1* type II collagen, *E2* estradiol, *ER* estrogen receptor, *TGFβ* transforming growth factor beta, *ALK5* activin-like kinase 5, *SOX9* sry-type high-mobility-group box transcription factor 9, *ACAN* aggrecan, *VEGF* vascular endothelial growth factor, *OPN* osteopontin, *OC* osteocalcin, *ALP* alkaline phosphatase, *SIRT1* silencing information regulator 2 related enzyme 1, *AMPK* AMP-activated protein kinase, *mTOR* mammalian target of rapamycin, *ULK* unc-51-like kinase, *ERRα* estrogen‑related receptor α, *GDF5* growth and differentiation factor 5, *GPER1/GPR30* G-protein coupled estrogen receptor, *PI3K* phosphatidylinositol 3-kinase, *AKT* protein kinase B, *NF-κB* nuclear factor kappa-B, *FOXO3* forkhead box O-3, *IL1* interleukin 1, *ASIC1a* acid-sensing ion channel 1a, *ADAMTS5* a disintegrin and metalloproteinase with thrombospondin motifs 5, *COX2* cyclooxygenase-2, *iNOS* inducible nitric oxide synthase, *TNFα* tumor necrosis factor α, *MAPK* mitogen-activated protein kinases, *miR-203* microRNA-203

Joint tissue biology is heavily influenced by estrogen, which helps to regulate the expression of key signaling molecules and their activity in several distinct pathways. Molecularly, the ERs are transcription factors that bind to the DNA either directly or indirectly; they are then able to signal through one of four pathways, three ligand-dependent and one ligand-independent [124, 125]. Increasing evidence indicates that interaction between estrogen and ERs as well as related signaling pathways contributes to sex-dependent variation in cartilage adaptation (Fig. 1).

### Wnt signaling pathway

During cartilage development and regeneration, Wnt14 is upregulated [126]. By binding to cell surface frizzled receptors (FZD) and low density lipoprotein receptor-related protein (LRP) co-receptors [9], Wnt14 inhibits β-catenin phosphorylation by glycogen synthase kinase 3 (GSK3). The unphosphorylated β-catenin then travels into the chondrocyte nucleus to act as a transcription factor [9], stimulating tissue breakdown typically consistent with the OA phenotype [127–129]. Since β-catenin is found in higher concentrations in OA tissue, the promotion of β-catenin degradation protects cartilage from damage [127, 128]. Interestingly, E2 upregulates the expression of the *SOST* gene, which codes for an inhibitor of Wnt14 called sclerostin [130]. By inhibiting Wnt14, β-catenin gets phosphorylated and tagged for degradation. Though E2 promotes sclerostin expression, this Wnt inhibitor is still found in higher concentrations in males than females, which may be a factor in the increased clinical incidence of OA in females [60]. Evidence indicates that DHEA decreases the expression of β-catenin [131] and inhibits *MMP13* expression and increases *TIMP1* and *COL2A1* expression in IL1β-induced rabbit chondrocytes [95]. Following treatment with DHEA after the transfection of β-catenin, rabbit chondrocytes showed significantly elevated expression of *MMP13* and depressed expression of *TIMP1* and *COL2A1*; meanwhile, after inactivating Wnt/β-catenin signaling with DKK1, the expression of *MMP3*, *MMP13*, and *TIMP1* were suggestive of enhanced protective effects of DHEA [131].

The β-catenin signaling pathway is activated by chronic dysregulation of circadian rhythm due to downregulation of brain and muscle ARNT-Like 1 (BMAL1) [132]. BMAL1 is a protein that helps generate circadian rhythms in cartilage; dysfunctional BMAL1 in OA results in increased expression of β-catenin, MMP3, MMP13, ADAMTS4, and subsequent cartilage degeneration [132]. Circadian rhythm dysregulation is also associated with dysregulation of TGFβ signaling in chondrocytes, which is an essential signaling pathway for cartilage homeostasis [133]. Though the exact mechanism is unknown, murine castration studies indicate that estradiol is an important factor for the modification of circadian rhythms during development in both sexes [134]. Therefore, alterations in estrogen level are a likely influence of OA development due to circadian rhythm dysregulation, especially during menopause when estrogen levels decrease significantly [134].

### TGFβ signaling pathway

Like the Wnt pathway, cartilage development and maintenance are highly regulated by TGFβ signals. TGFβ signals through type II receptors that recruit and subsequently activate type I receptors. Two main types of type I receptors, activin-like kinase 1 (ALK1) and ALK5, exist in cartilage and result in contrasting outcomes. Activation of ALK1 leads to the stimulation of terminal hypertrophic differentiation, characterized by increased production of *COL10A1*, *MMP13*, *VEGF*, *OPN* (osteopontin), *BGLAP* (osteocalcin), and *ALP* (alkaline phosphatase). On the other hand, E2 upregulating the expression of ALK5 results in the inhibition of hypertrophic differentiation and type II collagen and aggrecan production and maintains the quiescent stage of chondrocytes [9, 135, 136], indicating that E2 promotes chondrocyte development and homeostasis through the TGFβ pathway [75]. In OA, cartilage typically has a dramatic reduction of ALK5 receptors, indicating the protective role of E2 against cartilage degradation [9, 136].

This signaling pathway interacts with other signaling molecules such as BMPs and transcription factors such as HIFs. BMP7 exhibits both anabolic and anti-catabolic effects on cartilage. For example, BMP7 induces the production of ECM to help protect against damage from IL1, IL6, and fibronectin fragments; BMP7 is also involved in the preservation of chondrogenic potential [9]. On average, males consistently have higher BMP7 expression in cartilage, which is consistent with the lower incidence of OA in males versus females [137]. BMP2 exhibits both anabolic and catabolic effects on cartilage by inducing type II collagen and aggrecan production, promoting proteoglycan synthesis, and elevating MMP13 expression. BMP2 is typically elevated in cartilage injury or OA, suggesting that its primary role is likely regulation of MMP13. HIF1α and HIF2α are also elevated in degenerative conditions, but the two transcription factors have contradicting functions. Elevated HIF1α plays a compensatory role in damaged cartilage, as it promotes transcription of type II collagen and aggrecan. HIF2a, contrarily, increases the expression of MMP13 and ADAMTS4 for a catabolic effect. E2 downregulates *HIF1a*, *RUNX2*, and *BMP2*, all of which help maintain cartilage integrity [138–140]. In pathologic conditions, such as OA, the usual hypoxic conditions of articular cartilage are exacerbated, which stimulates the increased expression of HIFs [140]. Therefore, *HIF1a* is overexpressed in OA cartilage, leading to discoordination of type II collagen and aggrecan production, since HIF1α indirectly regulates *COL2A1* transcription [140]. E2 helps limit this discoordinate expression by reducing the expression of *HIF1a* in pathologically hypoxic conditions [140]. Similarly, the downregulation of BMP2 limits overexpression of *COL2A1* and *ACAN* genes while also reducing BMP2-induced expression of *MMP13* [9]. *RUNX2* coordinating with TGFβ signaling via ALK1 phosphorylation is typically upregulated in OA, so limiting its expression also reduces levels of *MMP13*, *COL10A1*, and *VEGF*, indicating hypertrophic differentiation and cartilage damage [9, 139].

### Notch signaling

Notch signaling pathways, highly conserved in mammals, require careful regulation for maintenance of healthy cartilage. Downstream, Notch upregulates expression of MMP13 while downregulating the synthesis of type II collagen. Therefore, Notch signaling is chondrodestructive in nature. This mechanism was demonstrated by an experimental mouse model in which one group received treatment with a γ-secretase inhibitor, an inhibitor of Notch, while the other group did not. In the group that did not receive the γ-secretase inhibitor, the mice exhibited greater severity of articular cartilage degeneration due to upregulated MMP13 and decreased synthesis of type II collagen [141]. Estrogen has been shown to upregulate Notch1, indicating a connection between female sex hormones and a chondrodestructive signaling pathway [142]. Further exploration of the relationship between biological sex and cartilage-specific Notch signaling is needed to better determine the specific mechanisms and interactions, as four types of Notch proteins exist, each with unique functions.

### Fibroblast growth factor

FGFs are important growth factors involved in the development and maintenance of articular cartilage. FGF18 serves an anabolic role in cartilage, acting as an inducer of chondrocyte proliferation and ECM synthesis. In a murine model, injection of FGF18 in OA joints resulted in increased cartilage formation [143]. Unlike FGF18, the exact role of FGF2 in cartilage maintenance is unclear; FGF2 seems to serve a combination of anabolic and catabolic functions. In the presence of existing cartilage defects, FGF2 was found to have a regenerative effect much like FGF18 [144]. However, while FGF2 effectively induced proliferation in these studies, it failed to induce ECM production [145]. This growth factor was also found to upregulate several metalloproteinases, such as MMP13, ADAMTS4, and ADAMTS5, leading to the stimulation of pro-inflammatory cytokines and the inhibition of anabolic molecules, such as BMP7 [9]. Since BMP7 is typically expressed at higher concentrations in males, male cartilage may be better equipped to override the inhibitory effects of FGF2 [137]. While limited data exist regarding the relationship between sex hormones and FGFs in cartilage, a study of bovine ovarian tissue concluded that FGF18 decreased the synthesis of estrogen and progesterone production, indicating a negative correlation between female sex hormones and FGF18 in female reproductive organs [146]. Whether this effect is limited to ovarian tissue warrants further exploration.

### Retinoic acid

Retinoic acid is well-known to influence chondrocyte-specific gene expression through interaction with retinoic acid receptors (RARs) in the nucleus [147]. Vitamin A metabolites act as ligands for RARs and are found to be elevated in OA patients [148]. As observed in a recent in vitro study, activated RARs have an inhibitory effect on chondrogenesis, especially RARα and RARγ [147]. Specificity protein 1 is a transcription factor present in chondrocytes that can form complexes with ERs, inducing the activation of RARα [125]. Therefore, the presence of ERs in chondrocytes directly relates to inhibition of chondrogenesis [125].

### Cellular energy and survival related pathway

E2 loss correlates with the increased incidence of knee and hip OA. Interestingly, in rat OA model chondrocytes, E2 mediated activation of PI3K (phosphoinositide 3-kinase)/AKT (protein kinase B) markedly stimulates cell proliferation [149]. Through this signaling, administration of E2 also elevates proliferation and viability of ATDC5 chondrocytes [150]. High expression levels of GPER1/GPR30 in the hypertrophic zone have been indicated as regulating longitudinal bone growth as the expression decreases during puberty [151]. GPER1/GPR30 also plays a role in chondrocyte proliferation and has been demonstrated as required for a normal estrogenic response in the growth plate [152].

Typically involved in ECM synthesis, the PI3K/AKT pathway is downregulated in human OA cartilage or in OA-like chondrocytes exposed to IL1 or TNFα [153, 154]. Chondrocyte apoptosis is negatively regulated by PI3K/AKT signaling through inhibition of NF-κB [155]. 17β-E2 could function through the PI3K/AKT/FOXO3 (forkhead box O-3) pathway by downregulating MMP3 expression and preventing ECM degradation [156]. A key suppressor of autophagy, the mTOR pathway is regulated through upstream PI3K/AKT and AMPK (adenosine 5′-monophosphate-activated protein kinase) pathways [157]. In OA cartilage, upregulated mTOR signaling has been shown to lead to increased chondrocyte apoptosis and decreased autophagy-related gene expression; as such, cartilage-knockdown of mTOR reduces apoptosis and upregulates autophagy, shifting cartilage homeostasis in mice [158]. Furthermore, mTOR-regulated autophagy is linked to the inflammatory response, serving as an integral link between inflammation and autophagy in OA pathogenesis.

AMPK is critical to maintaining cell energy metabolism and survival. Given their reciprocal enhancement of each other’s activity, silencing information regulator 2 related enzyme 1 (SIRT1) and AMPK interact closely to regulate energy, metabolism, and aging [159]. In OA chondrocytes, pharmacologic AMPK activation not only improved mitochondrial biogenesis and function but also stimulated SIRT1-peroxisome proliferator-activated receptor gamma (PPARγ) coactivator-1α (PGC1α) signaling, ultimately delaying chondrocyte aging [160]. One study showed that SIRT1 and mTOR play a role in regulating cell aging by adjusting the autophagy function [161]. Specifically, SIRT1 restored oxidative stress-induced autophagy impairment and improved embryonic stem cell (ESC) survival by blocking the mTOR pathway [162]. In contrast, SIRT1 inhibition activates the mTOR pathway, resulting in autophagy injury [163]. In a mouse OA model, E2 inhibited the mTOR pathway via activating ERK, promoting chondrocyte autophagy to protect AMPK mutant mice from OA [164]. In vitro*,* E2 given to ATDC5 chondrocytes at a pharmacological concentration is capable of inducing SIRT1 expression through the AMPK/mTOR pathway in mitophagy [165]. These data culminated in a new appreciation for 17β-E2 signaling in OA and point to the SIRT1-mediated AMPK/mTOR signaling pathway as a potential target for OA therapy.

### Acidic environment and cellular inflammation related pathways

In vivo, there is increased expression of ASIC1a co-localized with NF-κB expression in articular cartilage of rat adjuvant arthritis [166]. NF-κB signaling pathways are activated by IL6, IL1β, and TNFα signaling, leading to ASIC1a upregulation. Extracellular acid activation of ASIC1a could exacerbate the TNFα- and IL1β-mediated impact on ECM metabolism by increasing *MMP3*/*MMP13* and *ADAMTS5* mRNA expression in articular chondrocytes [166]. Extracellular acidification also increases intracellular Ca^2+^ influx, culminating in articular chondrocyte apoptosis, autophagy, pyroptosis, and necroptosis [167].

17β-E2 can increase the mRNA and protein expression levels of ERRα, which in turn led to an increase in *SOX9*, *GDF5*, and *CYP19A1* during in vitro mandibular condylar chondrocyte cultivation [55]. Additionally, estrogen treatment activates the AMPK/ULK1 (unc-51-like kinase 1) signaling pathway, which was abrogated by ERRα-silencing. 17β-E2 has been linked to ASIC1a protein degradation through the ERRα, resulting in protection against acidosis-induced cytotoxicity in chondrocytes [80]. The estrogen-mediated downregulation of ASIC1a expression was mitigated by methyl-piperidino-pyrazole, an inhibitor of ERRα, suggestive of the involvement of ERRα in the estrogen regulating expression of ASIC1a. AMPK–ULK1 signaling activation promotes protein degradation of ASIC1a by the autophagy–lysosome pathway [168]. DHEA reduced *COX2* (cyclooxygenase-2) and *iNOS* (inducible nitric oxide synthase) gene expressions [169]. These findings all point to the role of estrogen in promoting the autophagy–lysosome pathway-dependent degradation of ASIC1a and protecting against acidosis-induced cytotoxicity, which is thought to be influenced by the ERRα–AMPK–ULK1 signaling pathway [168].

### MicroRNA related signaling pathway

In the postmenopausal rat model, miR-203 has been suggested to have critical involvement in OA onset and worsening, indicating that inhibiting the miRNA may reduce cartilage degradation [170]. MiR-203 has been shown to increase cellular inflammation and injury. IL1β stimulation increased miR-203 expression, which inhibited ERα expression and decreased *ACAN* and *COL2A1* [171]. This finding indicates that miR-203 is especially critical in estrogen deficiency and ERα instability-induced OA [171]. Interestingly, estrogen treatment increased miR-140 level and suppressed MMP13 expression in human articular chondrocytes; miR-140 knockdown mitigated this inhibitory influence of estrogen; further, the estrogen/ER/miR-140 pathway inhibited IL1β-induced cartilage matrix degradation [76]. In idiopathic condylar resorption (ICR), an aggressive form of OA in adolescent female patients, the E2-miRNA-101-3p–HAS2 pathway has been considered important. It has been theorized that E2 targets miRNA-101-3p in synovial fibroblasts of ICR patients, which regulates HAS2 expression [172]. The connection between estrogen and miRNAs presents a sex-related mechanism that could provide a more specific treatment approach for OA.

## Regeneration and prevention

While sexual dimorphism has been well-documented in cartilage degeneration, cartilage regeneration may also exhibit variations between males and females. Sex-dependent differences in stem cell regenerative capacity must be considered when evaluating the effectiveness of OA treatment options. This section will review current findings on how sex influences the outcomes of cartilage regeneration therapy.

### Surgical methods

Currently, the most common form of cartilage repair is surgical intervention. While there are advantages to utilizing surgical methods, such as microfracture to treat cartilage defects, these practices are invasive and often lead to poor clinical responses, such as fibrocartilage formation [173]. Interestingly, current responses to surgical techniques for cartilage repair exhibit observable sex differences; however, further investigation is still needed to best apply this information in practice.

Microfracture is a procedure in which holes are drilled into areas of a cartilage defect in an attempt to circulate the healthy MSCs from deep within the cartilage to the surface, leading to cartilage regeneration [174]. While this procedure is considered less invasive than traditional surgical implantation, it can only be performed for small defects and thus has limited applicability [174]. Notably, microfracture does have some success in alleviating chondral degradation [174]. Males tend to have better microfracture outcomes than females, scoring higher on assessments for symptom relief and functional improvement [175]. Compared to female patients, more males also undergo microfracture treatments, along with chondroplasty and osteochondral allografts [176].

Autologous chondrocyte implantation (ACI) is a technique that harvests chondrocytes from a patient’s intact articular cartilage to be implanted at the patient’s site of defect or degeneration. This procedure is not ideal, as it disturbs healthy cartilage and may lead to donor site morbidity. However, some positive outcomes have been recorded, including pain reduction, tissue repair, and improvement of function [177]. Studies show that males typically display better responses to ACI treatment than females [177–179]. Compared to males, females who underwent ACI were found to have a higher rate of postoperative revision or arthroplasty [178, 180]. In a study that followed patients after ACI surgery, results revealed that defect location affects regeneration in females but not in males; males with defects on the femoral condyles had much better postoperative results than females with defects in the patellofemoral compartment [181]. Despite the documented sex differences in procedure results, there is still dispute regarding the significance of these disparities. Several studies suggest a lack of significant difference in ACI outcomes between males and females [182, 183]. These studies indicate that ACI has the potential to benefit both sexes indiscriminately and that sex does not influence the success of ACI [184].

Autologous matrix-induced chondrogenesis (AMIC) is a cartilage defect treatment that combines techniques of both ACI and microfracture, inserting a collagen scaffold and fibrin glue over the hole produced by microfracture [185]. This sort of treatment results in better cartilage regeneration for males than females [185]. However, recent studies reported conflicting results in which no significant differences in postsurgical prognosis were found between sexes, suggesting that both males and females have the same potential for successful AMIC outcomes [186, 187]. Since females tend to have worse preliminary defects, sex-related repair outcomes may falsely appear to have observable differences [184]. However, when compared over a long-term postoperative period, males and females exhibit similar levels of healing, especially when comparing younger populations [184].

### Sex hormone treatment

Since cartilage degeneration is closely linked to hormonal influences, as previously mentioned, sex hormone therapy has the potential to help prevent degeneration and enhance regeneration (Table 2). Studies have shown that estrogen and estrogen receptors may play a functional role in chondrocytes [188, 189]. ERs on female chondrocytes have higher estrogen affinity than those on male chondrocytes [190]. It is theorized that this observation may contribute to the increased female incidence of OA compared to males. Levels of estrogen decrease after menopause, and accordingly, OA becomes more prominent in postmenopausal females. As such, modulation of ERs may help treat collagen degradation [6].

**Table 2:** Impact of sex hormones on cartilage regeneration

Hormones	Study design	Treatment	Analysis	Results	Implications	References
T/DHT	In vitro; murine ESCs	Cultured with T, DHT, or NT at each of the following concentrations: 10^–6^ M, 10^–8^ M, and 10^–10^ M in ethanol	IHC for androgen receptor, qPCR, cell colorimetric assays, Western blot	T had no significant effects on proliferation, while DHT marginally inhibited proliferation of ESCs; NT significantly increased ESC proliferation	ESC proliferation from blastocyst is independent of androgen effects	[207]
T	In vitro; human IVD cells and BMSCs	Differentiation in the presence or absence of T (10 ng/mL)	PCR, immunoblotting, IHC	Male IVD cells exposed to T exhibited increased aggrecan and types I and II collagen expression compared to control; T had no effect on female IVD cell chondrogenesis or BMSC chondrocytes from either sex	T increases ECM production in male IVD cells during differentiation	[92]
	In vivo; male rabbit growth plate chondrocytes	ORX versus non-ORX male rabbits	Soft radiography, IHC, quantification of caspase-3 and PCNA expression	At weeks 8–15 for ORX group, proliferation was decreased in quantity and displayed a narrowed proliferating zone compared to control	T accelerates growth and maturation of the epiphyseal plate and stimulates proliferation in males	[206]
	In vitro; rat costochondral growth zone and resting zone chondrocytes	Incubation with various doses of T: 10,100, and 1000 ng/mL	Thymidine incorporation, cell quantity, ALP activity, and collagen production	T inhibited cell number and dose-dependent decrease in thymidine incorporation of male growth zone and resting zone chondrocytes; cell quantity and thymidine incorporation unaffected by T in female rat cells; only significant change in ALP activity was an increase in ALP activity in T-treated growth zone male chondrocytes; T stimulated production of collagen in male growth zone and resting zone chondrocytes but T did not affect female cell collagen production	T primarily influences male chondrocytes and induces osteoblastic and chondrogenic differentiation	[197]
	In vivo; human patients with severe knee OA	Unilateral TKR	Serum T levels, knee radiograph, and WOMAC pain/function analysis 6–8 weeks after surgery	On the operative knee, higher T levels were associated with less pain in both sexes; in the non-operative knee, higher T in women was associated with less disability	T is positively correlated with less pain after TKR in both sexes and less disability in women without TKR	[196]
E2	In vitro; human ASCs	Cultured with E2 (concentration of 10^–8^ M) or without E2	RT-PCR	E2 exposure led to inhibition of *COL2A1* expression and reduction of *ACAN* expression	E2 may have a negative impact on human ASC chondrogenesis	[216]
	In vitro; human BMSCs	Cultured with E2 (concentrations ranging from 10^–11^ M to 10^–8^ M) with or without ER inhibitor	Histology, IHC, quantification of type II collagen, sulfated GAGs, type X collagen, and MMP13	E2 treatment resulted in a reduction of type II collagen and enhancement of type X collagen and MMP13 expression	E2 suppresses chondrogenesis of human BMSCs	[215]
	In vitro; murine ESCs	Incubated in E2 (0–10^−6^ M for 8 h) or at 10^–9^ M for times ranging from 0 to 12 h	ALP staining, immunofluorescence, thymidine incorporation, BrdU incorporation, RT-PCR, Western blot	E2 treatment significantly increased thymidine incorporation, BrdU incorporation, and cell number	E2 stimulates proliferation of mouse ESCs	[208]
	In vitro; mini-pig BMSCs	Cultured in E2 at 0 M, 10^–6^ M, 10^–8^ M, 10^–10^ M, 10^–12^ M, or 10^–14^ M	Surface marker analysis, ALP, MTT proliferation assay, β-galactosidase staining, TUNEL staining, differentiation/cytochemical staining, RT-PCR	E2 increased proliferation in female BMSCs, with a dose of 10^–12^ M optimizing proliferation. E2 also increased proliferation in male cells to a lesser degree that is still significant; E2 at 10^–12^ M inhibited cellular senescence in BMSCs from both sexes; E2 did not significantly influence chondrogenic differentiation; E2 increased adipogenic differentiation ability in male BMSCs and osteogenic ability in female BMSCs	E2 improves cellular senescence and proliferation in both sexes	[210]
	In vitro; human articular chondrocytes	E2 treatment ranging from 10^–11^ to 10^–7^ M	Thymidine incorporation, ALP, RT-PCR for ER expression	E2-treated cells from female donors exhibited an increase in thymidine incorporation and ALP activity in a dose-dependent manner; no changes in thymidine incorporation or ALP activity seen in male cells with E2 treatment; no sex differences in ER expression when normalized to GAPDH	E2 stimulates proliferation and promotes osteogenic differentiation of female chondrocytes	[190]
	In vitro; female menopausal versus non-menopausal monkey articular chondrocytes	Transfection with a reporter construct containing ER element/luciferase	RT-PCR, immunoblotting, IHC	Articular cartilage contained functional ERs from both menopausal and non-menopausal donors	E2 receptors are functional after menopause, indicating ERT is functional after menopause	[189]
	In vivo; female monkey model	OVX female monkeys with and without ERT	Ligand blotting and immunoblotting to analyze production of IGFBPs; RT-PCR analysis for PG synthesis	IGFBP2 and PG expression was significantly increased in monkeys with ERT	ERT in menopausal females is chondroprotective	[189]

*ASC* adipose-derived stem cell, *ALP* alkaline phosphatase, *BMSC* bone-marrow-derived stem cells, *BrDU* bromodeoxyuridine, *DHT* dihydrotestosterone, *E2* 17-ß estradiol, *ESC* embryonic stem cell, *ER* estrogen receptor, *ECM* extracellular matrix, *GAGs* glycosaminoglycans, *GAPDH* glyceraldehyde 3-phosphate dehydrogenase, *IGFBP* insulin-like growth factor binding protein, *IHC* immunohistochemistry, *IVD* intervertebral disc, *MMP13* matrix metalloproteinase 13, *MTT* 3-(4,5-dimethylthiazol-2-yl)-2,5-diphenyltetrazolium bromide, *NT* antiandrogen nilutamide, *OA* osteoarthritis, *ORX* orchiectomy, *OVX* ovariectomy, *PG* progesterone, *PCNA* proliferating cell nuclear antigen, *qPCR* quantitative real-time polymerase chain reaction, *RT-PCR* reverse transcription polymerase chain reaction, *TUNEL* terminal deoxynucleotidyl transferase dUTP nick end labeling, *T* testosterone, *TKR* total knee replacement, *WOMAC* Western Ontario and McMaster Universities Osteoarthritis Index

Kinney et al. found that chondrocytes from healthy females respond to E2 treatment; however, a lack of response to E2 is observed in males [190]. Another study done on a murine model similarly confirmed estrogen’s chondroprotective effect in female mice but not in male mice [111]. Oral and transdermal E2 treatment resulted in a decrease in levels of urine CTX-I and CTX-II in women; since urine CTX is a product of cartilage degradation, this finding supports estrogen therapy as a promising treatment for women in the field of cartilage repair and regeneration [191]. Females with low serum estrogen concentrations are more likely to develop OA [192]. Therefore, menopausal women have a higher risk of cartilage degeneration because of estrogen decline [6]. In postmenopausal women, ERT seems to have a benefit in increasing cartilage thickness [193]. In an in vivo study, postmenopausal women having taken ERT for more than 5 years were observed to have more articular tibial cartilage than women without hormone replacement [193]. The Framingham Study also observed preventative and protective results against cartilage degradation in postmenopausal women on ERT; participants on ERT experienced less OA progression compared to the control, indicating cartilage protection resulted from estrogen supplementation [194]. ERT was also found to increase expression of IGF binding protein 2, proteoglycans, and collagen in articular cartilage, demonstrating some of the chondroprotective mechanisms of this therapy [189]. However, risks of hormone replacement may outweigh the benefits [195]. Therefore, additional therapies should continue to be considered for OA in postmenopausal women.

Sex steroids may help enhance the chondrogenic potential of human chondrogenic progenitor cells (CPCs) in a sex-dependent manner, improving the regeneration capacity of late-stage OA tissue [91]. Koelling and Miosge found that SOX9 was highly expressed in the CPCs from women treated with E2 and CPCs from men treated with testosterone; CPCs from women responded better to steroid treatment, which may be explained by the higher concentration of sex hormone receptors in females than males [91].

Unlike the strong body of research on ERT, not as much information exists on sex variation regarding testosterone replacement therapy. One study assessed the association between OA pain progression and total serum testosterone levels among patients who had undergone total knee replacement surgery and found that both sexes reported less pain with higher testosterone levels; interestingly, all females that reported less disability had higher levels of serum testosterone [196]. These findings suggest that testosterone levels are positively correlated with OA improvement in both sexes. However, a study led by Alessandro Bertolo concluded that male IVD cells exposed to testosterone experience an increase in expression of aggrecan and types I and II collagen, while the female cells experience no effect, suggesting that testosterone only influences chondrogenesis for male cells [92]. Another in vitro experiment in which male and female rat chondrocytes were treated with testosterone yielded similar results; testosterone treatment increased collagen production in male cells but not in female cells [197]. Current evidence points to testosterone being positively associated with cartilage health in males, but its role in females is less certain. More research investigating the potential of testosterone treatment is needed to fully understand if it can play a meaningful role in cartilage repair.

An alternative approach to sex hormone therapy is using isoflavone, a soy phytoestrogen from legumes. Soybean isoflovane can have estrogenic effects on tissues [198]. In OVX rats, soybean isoflavone has been observed to limit cartilage degeneration [199]. Another study observed arctigenin, a dietary phytoestrogen, worked as a cartilage protector in human chondrocytes and mouse OA models [200]. Similarly, another phytoestrogen, daidzein, had anti-inflammatory and anti-oxidant effects in a rat OA model, especially when in combination with hyaluronic acid therapy [201]. In human chondrocytes, daidzein has had comparable results, having positive effects on ECM formation and pheotype regulation [202]. These studies demonstrate using phytoestrogen as a therapy method could be a promising approach to treat OA.

### Stem cell therapy

**Fig. 2:**
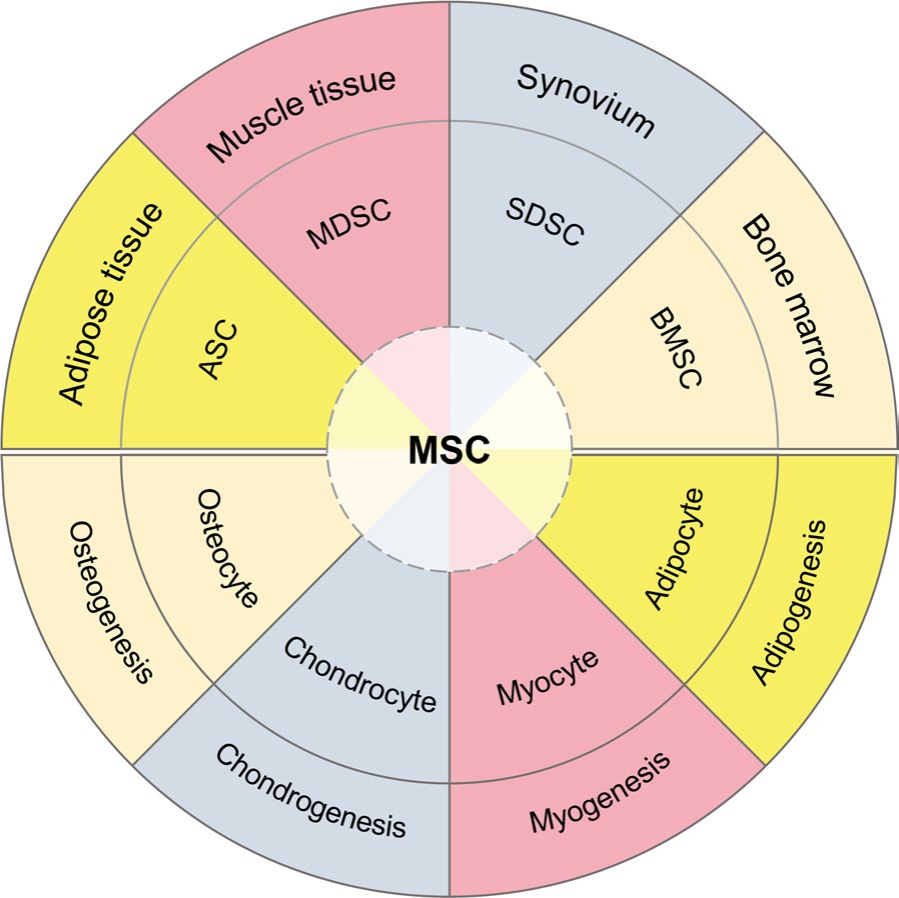
Origin and differentiation of MSCs including ASCs, MDSCs, SDSCs, and BMSCs. Upper hemisphere shows MSC origin and lower hemisphere shows MSC differentiation

Stem cell-based tissue engineering is a promising approach for repairing adult cartilage defects. Specifically, MSCs are at the forefront amongst stem cells for cartilage regeneration and repair given their high proliferation rate and chondrogenic potential [1]. MSCs can be found in different areas of the body, such as bone marrow, adipose tissue, synovium, and muscle, and can be differentiated into chondrocytes and other types of cells (Fig. 2) [203]. MSCs display sex differences in proliferation and differentiation [204], indicating that stem cell therapy can be enhanced by taking these differences into account.

#### Optimization of stem cell therapy

Given the integral role sex hormones play in cartilage maintenance and regulation, testosterone, DHT, and E2 have the ability to influence stem cell proliferation and differentiation and may be used to optimize stem cell therapy as a cartilage regeneration method. Studies on the influence of testosterone on stem cell proliferation have mixed results, indicating a need for further experimentation [205]. In a castration study, elimination of testosterone suppressed DNA synthesis of male bone-marrow-derived stem cells (BMSCs), implying testosterone has the capacity to stimulate proliferation [206]. However, testosterone was found to have the opposite effect in vitro*,* limiting the cell count of male chondrocytes [197]. In contrast, a study on ESCs concluded that testosterone had neither a positive nor negative influence on proliferation [207]. These mixed results indicate that cell type is a probable factor in the influence of testosterone on cell expansion. The same study also suggests that DHT is a more influential hormone on proliferation than testosterone, as DHT exposure demonstrated marginal inhibitory effects on stem cell proliferation [207]. The impact of testosterone on differentiation is more clearly established, with both in vivo and in vitro experiments concluding that testosterone exposure helps induce chondrogenic differentiation of BMSCs [197]. Furthermore, testosterone has been observed to increase ECM deposition in male IVD cells during differentiation, further supporting its role in promoting chondrogenesis [92]. While testosterone may stimulate chondrogenesis, sex of the stem cell donor must be considered, as testosterone likely has greater capacity to influence cells derived from male donors [197]. Further evaluation is needed to conclusively determine the impacts of androgens on proliferation and differentiation, but DHT may encourage proliferation, while testosterone promotes chondrogenic differentiation of BMSCs.

Contrary to male sex hormones, female sex hormones have remarkable evidence supporting their effects on stem cells, overall promoting proliferation and inhibiting differentiation. An in vitro study concluded that E2 promotes proliferation of ESCs by increasing cyclin D1, cyclin E, cyclin dependent kinase 4 (CDK4), and CDK2, therefore, promoting cell cycle progression [208]. Like ESCs, the proliferation rate of other stem cell types is also promoted by estrogen. The proliferation of BMSCs is mediated by estrogen in a concentration-dependent and sex-dependent manner [209]. Peak proliferation rate was produced by exposure of E2 at 10^–12^ M in female BMSCs and between 10^–8^ and 10^–12^ M in male BMSCs [209, 210]. Mouse adipose-derived stem cells (ASCs) also respond to E2 treatment by increasing proliferation rate through ERα [211]. In vivo, both BMSCs and ASCs isolated from female donors divide more rapidly compared to those from male donors, indicating that the effect of estrogen on proliferation is greater than that of testosterone at physiologic concentrations [212, 213]. Since menopause reduces estrogen production, resulting decreases in proliferative capacity of BMSCs contributes to the rise in incidences of OA and osteoporosis [214]. Estrogen hormone therapies may be considered for treatment options, but only for females, as only chondrocytes from female donors respond to E2 [190]. While estrogen stimulates stem cell proliferation, it has the opposite effect on differentiation. In vitro studies concluded that E2 has an inhibitory effect on chondrogenic differentiation of both ASCs and BMSCs [215, 216].

Similar to endogenous hormones, exogenous factors such as dietary metabolites also influence properties of stem cell growth and may be used to optimize stem cell therapies. Metabolites of Vitamin D impact differentiation, with 24,25-(OH)2D3 increasing the size of the hypertrophic zone of cartilage at the growth plate in both sexes [217]. 1-Alpha,25(OH)2D3 was found to induce expression of E2 in female, but not male, chondrocytes, which may be partially responsible for the chondroprotective role of Vitamin D in females [32]. While Vitamin D is generally chondroprotective in both sexes, estrogen promotes Vitamin D accumulation and upregulation of Vitamin D receptors, indicating it may have a greater impact in females [218]. Another metabolite impacting chondrogenic differentiation and proliferation is maternal β-hydroxy-β-methyl butyrate (HMB), a product of leucine metabolism. In vivo, prenatal HMB treatment of pregnant sows increased proteoglycan content in articular cartilage, especially in female offspring [219]. HMB treatment also increased IGF-I concentration, proliferation, and survival in both sexes of offspring. In HMB-pretreated female offspring, the temperature at which collagen denatured was observed to be significantly higher compared to that in the control group, suggesting that prenatal HMB treatment may yield female offspring with stronger, more resistant cartilage [219]. Male cartilage has a higher abundance of cross-linking proteins, such as lysyl oxidase-like protein 2 and fibulins, which likely contributes to the greater impact of HMB treatment on female offspring compared to males [220].

Other treatments yield sex-specific responses in stem cells as well, such as exposure to hypoxia and ROS. After undergoing lipopolysaccharide dose treatment followed by hypoxia treatment for 1 h, female-derived MSCs consistently produced more VEGF than male-derived cells and also exhibited a decrease in TNFα expression, while male donor cells did not [221]. These findings further support the notion that male and female stem cell properties differ and can be enhanced with more specialized cultivation techniques to advance stem cell therapy.

#### Sex differences in stem cell therapy

Currently, few studies distinctly focus on the potential differences between sexes in MSC cartilage regeneration. This review aims to summarize the overall sex-dependent trends and implications they have for future clinical applications. Human BMSCs have been identified as a good source for articular cartilage repair because of their great chondrogenic capacity [222]. Increasing research supports that human BMSCs display sexual dimorphisms in regenerative ability. Specifically, studies have pointed to male-derived BMSCs exhibiting greater chondrogenic potential than female cells [223]; male BMSCs decrease in chondrogenic potential with age, while female BMSCs do not [224]. However, a recent study found no substantial differences in adipogenic and chondrogenic differentiation potential of human BMSCs from healthy male donors as compared to healthy female donors [225].

Another promising cell candidate for stem cell-based therapy is muscle-derived stem cells (MDSCs). MDSCs display good chondrogenic potential and cartilage regeneration properties [226]. Like BMSCs, this cell type also exhibits variation in regenerative ability between male and female donors. An in vitro study revealed that human and murine MDSCs from male donors might have a greater chondrogenic potential than that of female donors [227]. Sex not only influences MDSC proliferation and rejuvenation potential, but also that of ASCs; ASCs from male donors also demonstrate greater chondrogenic potential compared to female donors [223, 228]. Overall, current studies point to donor sex as a major determinant of stem cell properties, conclusively establishing the need to disclose donor sex in all future studies. With this measure in place, sexual dimorphisms of stem cells will not be a confounding variable.

Interestingly, the sex of the treatment recipient has also been shown to influence treatment response. Some experiments have demonstrated that males respond better to stem cell treatment. For example, an in vivo experiment implanted BMSCs into human subjects with articular cartilage defects in the knee and found that outcomes were sex-dependent. Results revealed significant differences between sexes up to 10 years postoperatively, with males experiencing greater response to the method in terms of physical ability, repair, and healing after implantation [177, 179]. This finding may relate to the high incidence of OA in postmenopausal women with low estrogen levels which is linked to increased OA susceptibility and may hinder their ability to respond to treatment. Women also tend to develop worse cases of OA compared to men, which could also contribute to decreased responses to treatment [184].

## Discussion/conclusion

This review aims to highlight and summarize the sex differences in the field of cartilage repair and regeneration, specifically in regard to cartilage degeneration treatment with stem cell-based therapy. It is well-known that women have a higher likelihood of developing OA compared to men, but the mechanisms underlying this clinical finding are still being investigated. A decrease in estrogen following menopause seems to be an underlying trend in women with OA, leading to the conclusion that estrogen is a big factor in cartilage degradation and can be supplemented for treatment and prevention of OA in women. However, men may also develop cartilage degeneration. Though males do not respond to estrogen treatment like women, males have responded to testosterone treatment, indicating that androgens also play a role in OA. These distinctions in sex-based response to hormone therapies indicate the presence of variation in treatment options between sexes.

Biomarkers also are integral to the prevention of cartilage degeneration. Increasing evidence points to sex-dependent differences between cartilage biomarkers, suggesting that signs of OA in males and females present differently. Understanding the sex differences between biomarkers may lead to earlier diagnosis and the development of sex-specific treatment options.

In regard to stem cell therapies, the role of donor sex in MSC-based treatments is still being investigated. Currently, there is contradictory evidence, meaning further experimentation must be done to accurately determine the influence of donor sex on the regenerative potential of MSCs. Some studies conclude that male donors have healthier MSCs with more chondrogenic potential, making them more ideal candidates for stem cell treatment. However, other studies found no significant difference in the quality of MSCs between healthy male and healthy female donors.

The impact of recipient sex on cartilage repair method efficacy is also up for debate. In the case of BMSCs for stem cell treatment, males seem to experience better postoperative results than females. The difference in male and female responses to receiving stem cell treatment has not been extensively studied and needs more research. ACI treatment also seems to benefit males more, with females having less successful post-ACI outcomes. However, this finding may be skewed by the increased likelihood of women to have more severe cartilage defects than men going into these procedures, as there are some studies that show a lack of significant differences in the outcomes of surgical treatments.

This study focuses on sex differences at the molecular level and the implications on cartilage health and therapeutic strategies. Obesity and ethnicity factors in sex-related differences are not heavily discussed as they can be influenced by lifestyle and economic elements. However, there is merit for studying these factors, and future studies should focus on such elements to truly optimize clinical strategies for cartilage health.

In conclusion, there are clear sex-based variances in cartilage degeneration and regeneration, and the underlying mechanisms and exact effects still need further exploration. Additional research is critical to fully understand the role of sex in cartilage repair. Currently, most studies do not adjust their designs for sex variability and comparisons, resulting in a lack of statistical evidence on the influence of sex [2]. By fully understanding the sex differences in cartilage degeneration and regeneration, future studies can more intentionally select a target demographic. The development of sex-specific approaches to cartilage tissue engineering aims to provide more personalized and effective clinical treatments.

## Data Availability

Not applicable.
